# Bilateral Knee Dislocation in an Elderly Patient: From Vascular Emergency to Functional Recovery

**DOI:** 10.7759/cureus.102812

**Published:** 2026-02-02

**Authors:** Aziz Benakrout, Nada Belhachmi, Tarik Baadi, Ismail Aissa, Mustapha Bensghir

**Affiliations:** 1 Anesthesiology and Perioperative Medicine, Mohammed V Military Hospital, Faculty of Medicine and Pharmacy, Mohammed V University in Rabat, Rabat, MAR; 2 Vascular Surgery, Mohammed V Military Hospital, Faculty of Medicine and Pharmacy, Mohammed V University in Rabat, Rabat, MAR

**Keywords:** bilateral knee dislocation, computed tomography angiography, frailty, geriatric trauma, vascular emergency

## Abstract

Traumatic knee dislocations are rare but serious injuries associated with limb-threatening vascular compromise and a frequently poor functional prognosis. We report the case of a 70-year-old man with known dementia who sustained a high-energy road traffic accident resulting in bilateral knee dislocation. On admission, he was hemodynamically stable with preserved and symmetrical distal peripheral pulses. Initial radiographs confirmed bilateral dislocation, and computed tomography angiography of the lower limbs showed no arterial injury, particularly of the popliteal arteries. After pre-anesthetic assessment, bilateral closed reduction was performed under spinal anesthesia, followed by stabilization with trans-tibio-femoral screws within a multidisciplinary management pathway. The postoperative course was characterized by appropriate multimodal analgesia, early mobilization and physiotherapy, and pharmacologic thromboprophylaxis, allowing satisfactory functional recovery without vascular or neurologic complications. This case highlights the need for anesthesiologist-intensivists to balance the risk of vascular emergency, geriatric frailty, and the goals of rapid functional recovery.

## Introduction

Traumatic knee dislocation is defined as a complete loss of femorotibial congruence and is almost always associated with multiligamentous injury, involving rupture of multiple stabilizing ligaments [1]. Although rare, accounting for less than 0.02% of all musculoskeletal trauma, it typically follows high-energy mechanisms and carries a high risk of limb-threatening vascular compromise, particularly popliteal artery injury, which may result in acute limb ischemia, as well as neurologic and functional sequelae [2,3]. These potential complications require early recognition and coordinated multidisciplinary management. Bilateral traumatic knee dislocation is exceptional because it generally requires extreme traumatic forces and results in profound biomechanical instability, further increasing the complexity of clinical assessment and treatment. In frail older adults, this scenario poses additional challenges related not only to vascular evaluation but also to perioperative anesthetic management, geriatric frailty, and the need for rapid functional recovery, making it highly relevant to anesthesiologist-intensivists.

## Case presentation

**Figure 1:**
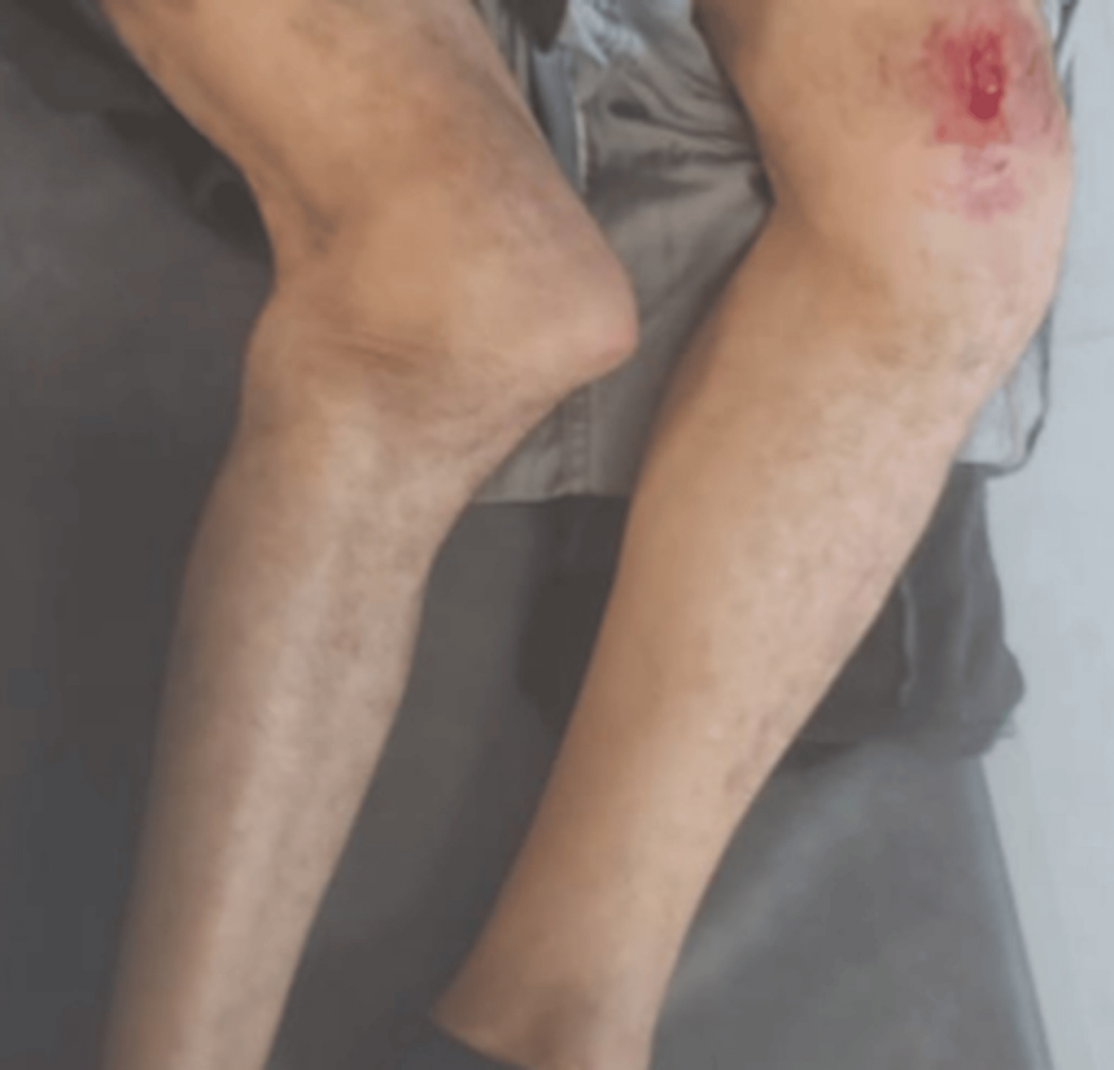
Initial clinical presentation (anterior view) showing bilateral knee deformity The patient is in the supine position. Both knees demonstrate marked deformity consistent with acute bilateral knee dislocation, with visible abnormal joint alignment and associated soft-tissue injury over the right knee

A 70-year-old man with a history of treated dementia and no other major known comorbidities was admitted to the emergency department after a high-energy road traffic accident involving a direct impact to both lower limbs. On arrival, he was awake and alert, with a Glasgow Coma Scale (GCS) score of 15/15, and was oriented in time and space relative to his baseline cognitive status. His chief complaint was severe bilateral knee pain with functional impairment of both lower limbs. Hemodynamic and respiratory parameters were stable: blood pressure 135/80 mmHg, heart rate 95 beats per minute, oxygen saturation 98% on room air, and respiratory rate 20 breaths per minute. There were no signs of respiratory distress or significant external bleeding. Musculoskeletal examination revealed marked deformity of both knees with severe pain on manipulation and complete functional impotence of the lower limbs. The knees appeared grossly unstable on clinical assessment, raising a strong suspicion of multiligamentous disruption. There was a superficial skin laceration over the right knee. Femoral, popliteal, dorsalis pedis, and posterior tibial pulses were palpable, symmetric, and of good amplitude. Distal motor function and sensory function were preserved, with no obvious peripheral neurologic deficit (Figure 1).

**Figure 2:**
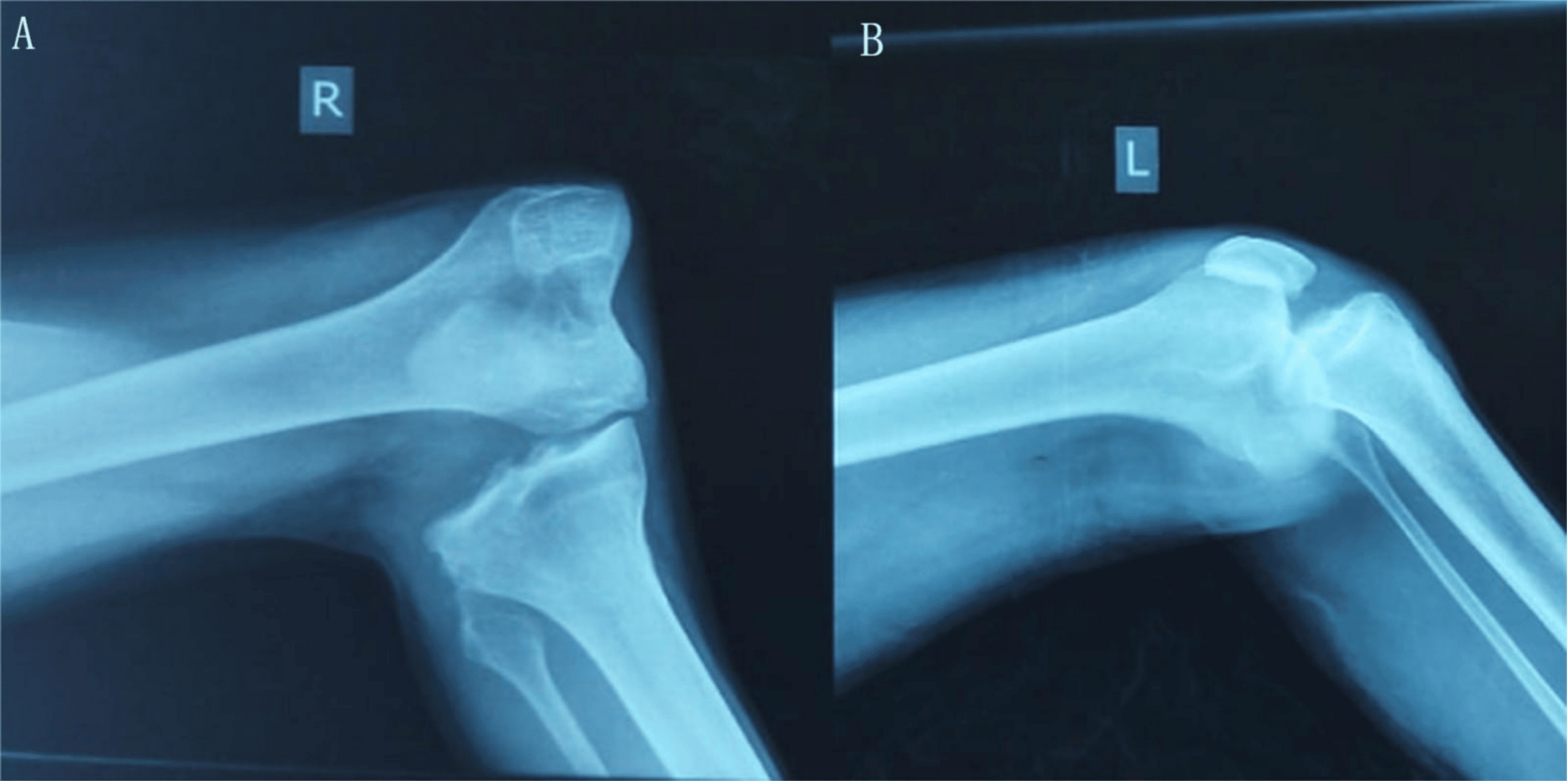
Bilateral knee dislocation on lateral radiographs Lateral radiographs of the right knee (A) and left knee (B) on admission, showing bilateral knee dislocation with complete loss of femorotibial congruence

**Figure 3:**
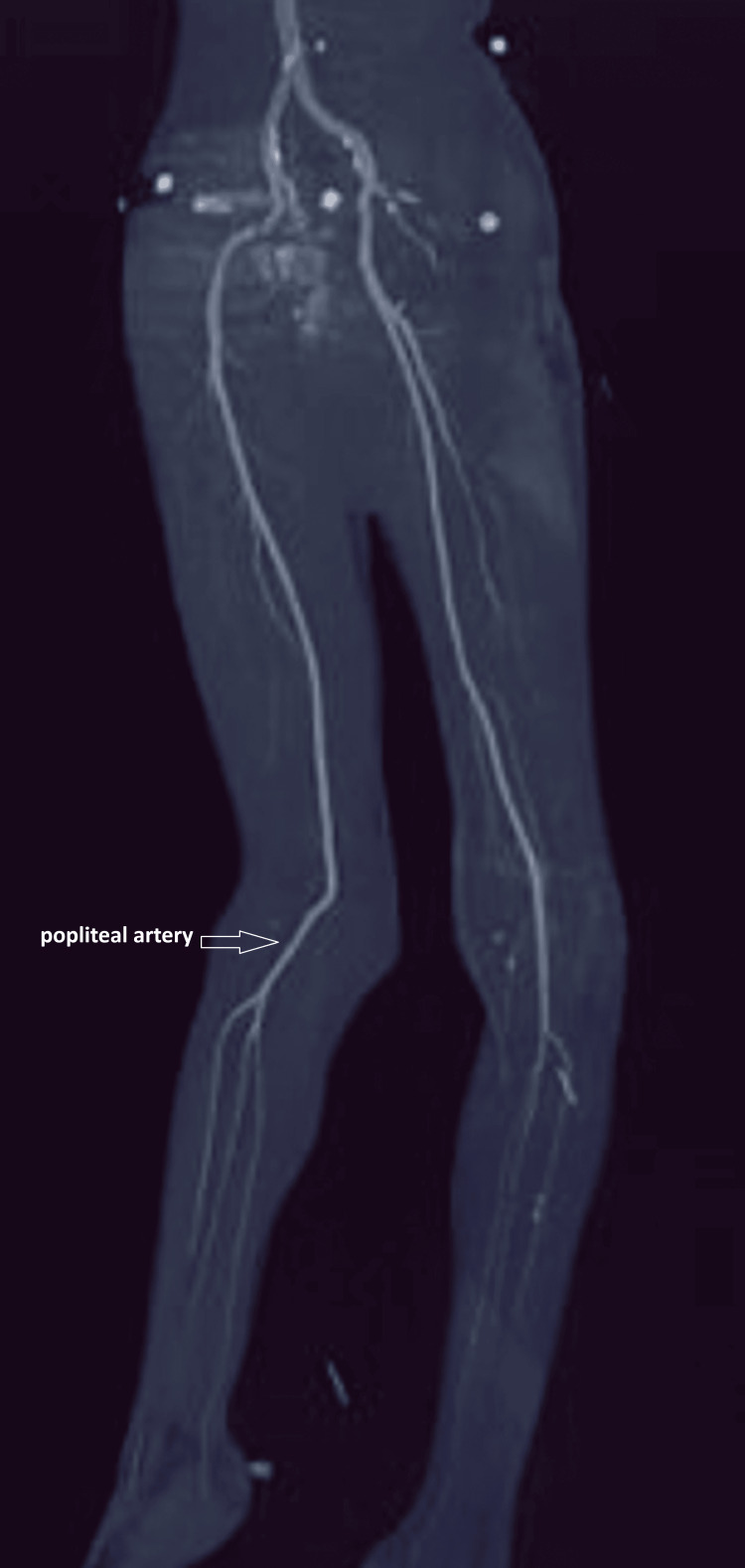
CT angiography of the lower limbs demonstrating preserved arterial patency despite traumatic knee dislocation The popliteal arteries are clearly visualized along their course, with no evidence of intimal injury, thrombosis, dissection, or contrast extravasation, highlighting that normal distal pulses do not exclude the need for vascular imaging in high-energy knee dislocations

Initial radiologic assessment was performed within the first hour after admission and included standard radiographs of both knees, which demonstrated bilateral dislocation with complete loss of femorotibial congruence (Figure 2). Whole-body trauma computed tomography did not reveal any associated visceral or osseous injuries. Given the high-energy mechanism and the known risk of occult vascular injury despite preserved distal pulses, emergent CT angiography of the lower limb arteries was performed and showed a patent vascular tree without thrombosis, dissection, or intimal injury, particularly at the tibio-peroneal trunks (Figure 3).

Initial emergency management consisted of standard resuscitation measures, intravenous paracetamol, and intravenous morphine administered in incremental boluses (1-2 mg every five minutes), titrated to achieve adequate pain control (target pain score ≤3/10 on a numeric rating scale), under close monitoring of respiratory rate, oxygen saturation, insertion of a large-bore peripheral venous line, and continuous monitoring of hemodynamic and respiratory parameters. Bilateral knee reduction was performed within approximately four hours of admission, after completion of the radiologic work-up.

Pre-anesthetic assessment focused on neurocognitive status, baseline functional autonomy, risk of malnutrition, and cardio-respiratory and renal function. Examination of the spine showed some lumbar stiffness but no contraindication to neuraxial anesthesia. Spinal anesthesia was chosen in preference to general anesthesia in order to limit hemodynamic fluctuations, avoid airway manipulation, and allow continuous neurologic assessment in this frail older patient with pre-existing cognitive impairment.

**Figure 4:**
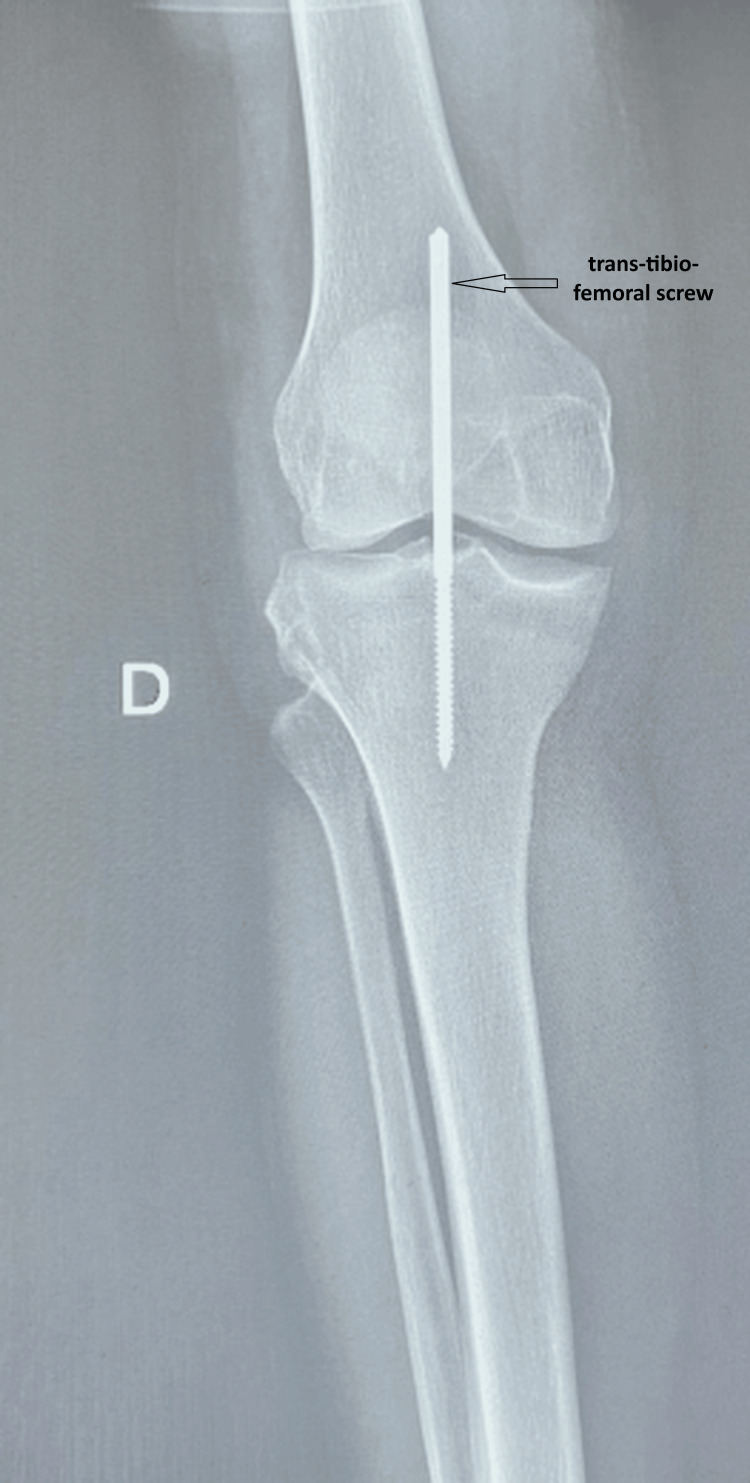
Standard radiograph of the right knee after reduction and stabilization with a trans-tibio-femoral screw

**Figure 5:**
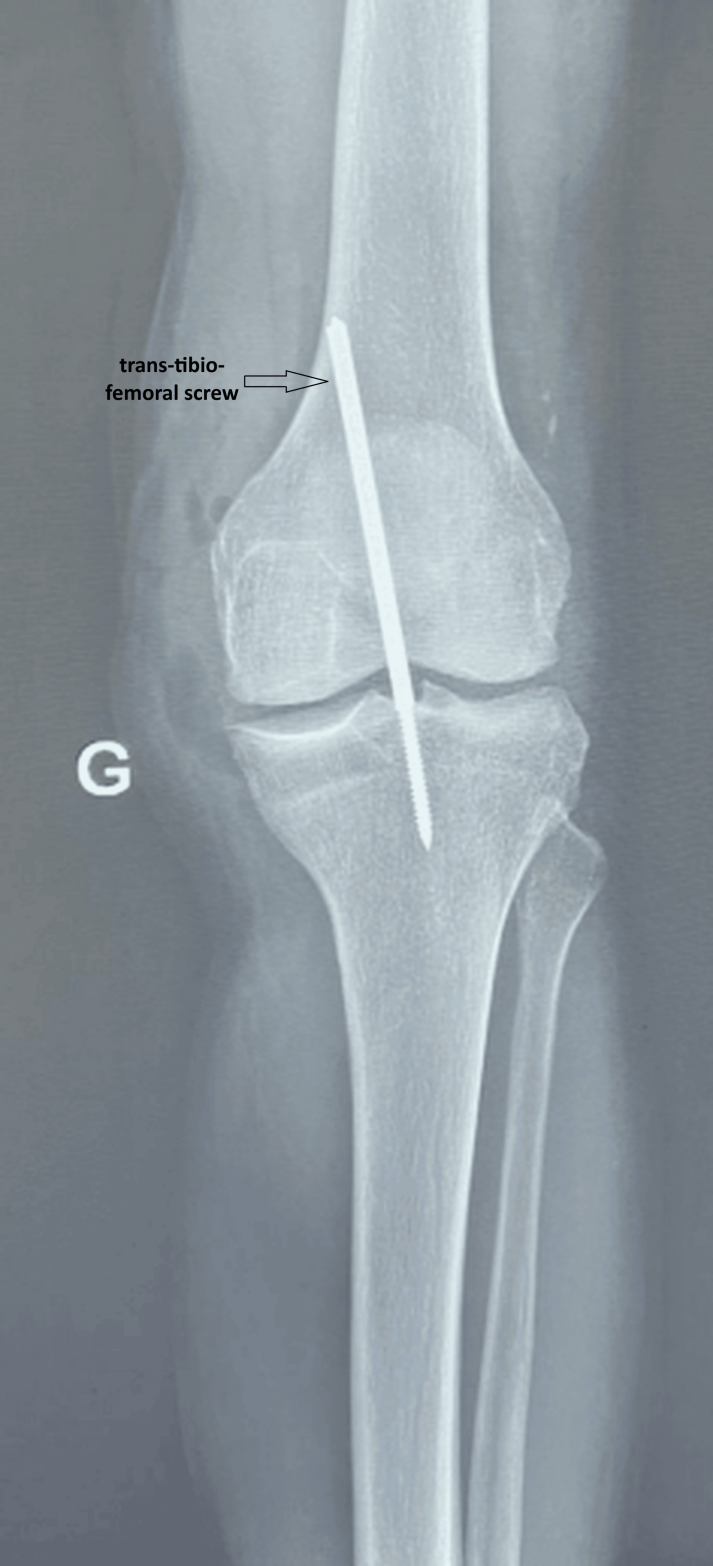
Standard radiograph of the left knee after reduction and stabilization with a trans-tibio-femoral screw

In the operating room, active warming was applied, and monitoring included continuous electrocardiography with ST-segment analysis, non-invasive blood pressure, pulse oximetry, and temperature. The large-bore peripheral venous line was maintained. Spinal anesthesia was performed with the patient in the sitting position, with careful positioning due to pain and joint stiffness. A fine needle was introduced at the L3-L4 interspace (identified clinically), and 7.5 mg of hyperbaric bupivacaine (0.5%) combined with fentanyl 25 µg was administered intrathecally. Sensory blockade was assessed clinically and reached T10 prior to incision. Bilateral orthopedic reduction of the dislocations was followed by stabilization with trans-tibio-femoral screws, chosen to provide immediate joint stability and allow early mobilization, rather than prolonged immobilization or external fixation, which were considered less suitable in this cognitively fragile patient (Figures 4, 5). The procedure lasted 60 minutes. Given age-related reduced physiological reserve, hemodynamic monitoring was closely maintained, and fluids were administered cautiously. Intraoperative fluid management consisted of 400 mL of crystalloids, with an estimated blood loss of 100 mL. No major intraoperative hemodynamic events occurred. At the end of surgery, before transfer, motor recovery was assessed as a Bromage score of 0 (Figures 4, 5).

In the post-anesthesia care unit, management relied on multimodal analgesia with scheduled intravenous paracetamol (1 g every six hours; maximum 4 g/day) and intravenous morphine administered in small incremental boluses (1 mg every five minutes), titrated to adequate analgesia. Pain was assessed regularly using a numeric rating scale (0-10) when self-report was reliable, and an ALGOPLUS® observational scale when assessment was limited by cognitive impairment, with titration targeting minimal pain behaviors and patient comfort while maintaining respiratory safety. This titrated opioid-sparing approach allowed us to avoid non-steroidal anti-inflammatory drugs because of their potential nephrotoxicity in older adults and to avoid nefopam due to its frequent neuropsychiatric adverse effects, particularly cognitive disturbances and agitation in the elderly. Close monitoring of neurologic status, urine output, temperature, and hemodynamic parameters was ensured. The patient was able to sit in a chair within the first 24 hours postoperatively and initiated early functional rehabilitation in collaboration with the physiotherapy team. Thromboprophylaxis with low-molecular-weight heparin combined with mechanical measures was administered.

At discharge, the patient was able to stand with assistance and begin assisted ambulation. Knee flexion was approximately 60 degrees bilaterally. The postoperative course was favorable, with no vascular or neurologic complications and no documented thromboembolic event. The patient was transferred to a rehabilitation unit for continued functional recovery. Follow-up in this report is limited to the early postoperative period; longer-term outcomes regarding ligamentous reconstruction, joint stability, and degenerative changes could not be assessed.

## Discussion

This report describes bilateral knee dislocation in a frail older adult following high-energy trauma, a scenario rarely reported in the literature. This injury is associated with major limb- and life-threatening complications, as well as substantial functional risk, and requires coordinated management by emergency physicians, anesthesiologist-intensivists, trauma surgeons, and radiologists [1,2].

Traumatic knee dislocation, which accounts for less than 0.02% of all musculoskeletal injuries, most often occurs in the context of multi-ligament knee injuries, for which management must be early and individualized according to the mechanism of injury, post-reduction stability, and patient-specific factors, particularly in the elderly [3]. In our case, internal fixation with trans-tibio-femoral screws was selected to ensure immediate stability and facilitate early rehabilitation, in contrast to external fixation or prolonged bracing, which may increase immobilization-related complications in frail patients.

Vascular complications are a major concern in knee dislocations, with the incidence of popliteal artery injury reaching 7-18% in some series, justifying heightened vigilance and repeated reassessment even when peripheral pulses are initially palpable [4]. Palpation of pulses alone may be misleading; therefore, a complete clinical examination should be combined with measurement of the ankle-brachial index and, in cases of doubt or high-energy mechanisms, lower-limb CT angiography to detect intimal lesions or occult thrombosis, as suggested in recent literature [5].

In the presence of acute limb ischemia, knee dislocation becomes a true vascular emergency, with an optimal revascularization window commonly estimated at less than 6-8 hours to limit the risk of amputation. This situation requires rapid organization of the patient pathway and close collaboration between vascular surgeons, trauma surgeons, and anesthesiologist-intensivists [6].

In older adults, anesthetic management falls within the concept of frailty, defined by decreased functional reserve and reduced capacity to adapt to surgical stress. Aging is accompanied by neurologic, cardiovascular, respiratory, renal, and musculoskeletal changes, as well as increased thromboembolic and fracture risk, which require a comprehensive preoperative assessment incorporating neuropsychological status, functional autonomy, nutritional status, and comorbidities, in line with the recommendations of the French Society of Anesthesia and Intensive Care. Regional anesthesia, particularly spinal anesthesia or peripheral nerve blocks, is often the preferred option in older adults when feasible, as it reduces exposure to general anesthetic agents, provided that constraints related to spinal osteoarthritis and stiffness are taken into account and that ultrasound guidance or fine needles are used. In our case, spinal anesthesia allowed gentle positioning, stable hemodynamics, and continuous neurologic monitoring [7].

Postoperative pain management in older adults is based on regular assessment using tools adapted to cognitive impairment, such as the Algoplus behavioral pain scale, and on multimodal analgesia favoring paracetamol and low-dose titrated opioids, with cautious use of agents such as nefopam and avoidance of nonsteroidal anti-inflammatory drugs to limit renal, gastrointestinal, and neurologic adverse effects [8].

Perioperative neurocognitive disorders, particularly postoperative delirium and postoperative cognitive dysfunction, are frequent in older adults and are promoted by pre-existing dementia, urgent surgery, and poorly controlled pain. Prevention relies on hemodynamic and respiratory optimization, maintenance of normothermia, minimization of sedative drugs, early replacement of glasses, hearing aids, and dentures, re-orientation in time and space, involvement of the family, and early rehabilitation [9].

Finally, polytraumatized older adults are at high risk of venous thromboembolism, warranting systematic thromboprophylaxis combining early mobilization, mechanical methods (stockings, intermittent pneumatic compression), and low-molecular-weight heparin adjusted to renal function, in accordance with recent recommendations and as implemented in our patient [10].

Postoperative outcomes were favorable in the short term, with early mobilization and recovery of assisted ambulation. However, the absence of long-term follow-up represents a limitation of this report, particularly regarding ligamentous reconstruction and the risk of post-traumatic osteoarthritis.

## Conclusions

Bilateral knee dislocation in the elderly is an exceptional trauma that should be approached as a potential vascular emergency until limb perfusion has been definitively secured. This case highlights the importance of a structured vascular assessment, including CT angiography when doubt persists despite preserved peripheral pulses, in order to reduce the risk of missed popliteal artery injuries and delayed diagnosis. This report also illustrates how preoperative identification of geriatric frailty and cognitive impairment can help tailor the anesthetic strategy, favoring spinal anesthesia, careful hemodynamic management, and early mobilization in selected patients. Optimized multimodal analgesia, appropriate thromboprophylaxis, and close postoperative surveillance were associated in this case with an uncomplicated early postoperative course and early functional improvement. The favorable short-term outcome observed supports the value of close multidisciplinary collaboration between anesthesiologist-intensivists, emergency physicians, trauma and vascular surgeons, radiologists, and physiotherapists. However, conclusions regarding long-term functional recovery and broader applicability must be interpreted with caution, as this report describes a single case with limited follow-up. Beyond its rarity, this case is intended to be hypothesis-generating and emphasizes the potential benefits of a coordinated, protocolized perioperative pathway in the management of frail elderly patients with limb-threatening knee injuries. Further studies are needed to better define optimal strategies and long-term outcomes in this growing population of geriatric trauma patients.
